# Development and Validation of a Culture-Sensitive Generic Health Literacy Scale in Turkish-Speaking Adults

**DOI:** 10.3928/24748307-20211208-01

**Published:** 2022-01

**Authors:** Şevkat Bahar Özvarış, Bahar Güçiz Doğan, Hande Konşuk Ünlü, Ozge Karadag, Nuri Doğan, Selahattin Gelbal, Sibel Sakarya

## Abstract

**Background:**

Improving health literacy has become one of the most important public health-related goals at the global level; however, there is no clear consensus on measurement of health literacy. Despite numerous health literacy scales available in Turkish, none of the existing scales was originally developed and validated at a national level.

**Objective:**

This study aimed to develop and validate a culturally appropriate original health literacy scale (HLS) to be used as a reference for the Turkish-speaking literate adult population in Turkey and abroad.

**Methods:**

Two multidisciplinary workshops with more than 20 experts were conducted and a large item pool was developed. The first and second draft of the scale were pre-tested with 20 and 150 adults, respectively, from different age groups and socioeconomic levels in Ankara. The validity and reliability study of the revised scale (110 items plus 20 self-efficacy statements) was carried out with a household survey of 2,411 adults in 12 randomly selected provinces from 12 Nomenclature of Territorial Units for Statistics Regions in Turkey. Explanatory and confirmatory factor analysis were performed. The fit indices were obtained. The item analysis was applied, and Cronbach's alpha statistics were obtained.

**Key Results:**

The scale was found to be both a valid and a reliable measurement tool to assess health literacy. Cronbach's alpha for two sub-dimensions (“disease prevention and health promotion” and “treatment and access to health services”) were 0.79 and 0.91, respectively. Construction validity indices were Root Mean Square Error of Approximation (RMSEA) = 0.043, Goodness of Fit Index (GFI) = 0.96, Normed Fit Index (NFI) = 0.95, and Adjusted Goodness of Fit Index (AGFI) = 0.95. The scale includes “self-efficacy” as an additional dimension (Cronbach's alpha = 0.83, RMSEA = 0.68, GFI = 0.94, NFI = 0.94, and AGFI) = 0.91).

**Conclusion:**

HLS is a valid and reliable measurement tool to assess health literacy of Turkish-speaking literate adults with a mixed (objective and subjective) assessment approach. **[*HLRP: Health Literacy Research and Practice*. 2022;6(1):e2–e11.]**

**Plain Language Summary:**

This study aimed to develop and validate a culturally sensitive original health literacy scale to be used as a reference scale for the Turkish-speaking literate adult population in Turkey and abroad. Study findings showed that HLS is both a valid and a reliable measurement tool to assess health literacy of Turkish-speaking literate adults.

As a complex concept, the definition of health literacy is highly context dependent. This complexity poses the challenge of making a standard definition of health literacy and developing tools that measure all its dimensions (Poureslami et al., 2017; Sørensen et al., 2012). In addition, current health literacy measures have been criticized for solely measuring reading and numeracy skills when a broader set of skills is necessary for making informed health decisions (Curtis et al., 2015).

In a systematic review of health literacy assessment tools by Altin et al. (2014), about one-third of the originally developed instruments used either a direct test of a person's abilities (objective measurement) or the elicitation of self-reported abilities (subjective measurement). The study found that almost all instruments applied a multidimensional measurement, and a majority used a mixed approach; however, according to the authors, “there is no clear indication of the demanded consensus on health literacy measurement” (Altin et al., 2014, p.10). Pleasant et al. (2011) concluded that there have been only minor developments among the measurement formats, even though the academic world was calling for new instruments.

When the scales in Turkish were examined, all of them were found to be adapted from scales developed in English. For example, the most widely used scales—Rapid Estimate of Adult Literacy in Medicine (REALM) (Davis et al., 1991), Newest Vital Sign (NVS) (Weiss et al., 2005), and Short Test of Functional Health Literacy (S-TOFHLA) (Baker et al., 1999)—were adapted to Turkish by different research groups (Eyüboğlu & Schulz, 2016; Ozdemir et al., 2010). However, Ozdemir et al. (2010) found that using two different scales revealed different health literacy levels for the same Turkish patient group studied. Other studies showed that cultural and linguistic differences also affect measurements, which sometimes give contradictory results (Weiss et al., 2005). In a study comparing migrant and host populations, Turkish migrants were found to have higher NVS scores than Dutch participants, whereas they scored lower in REALM when compared to the same host population (Fransen et al., 2011).

Two of the largest health literacy assessment studies in Turkey used measurement tools that were either adapted from or based on the framework of the European Health Literacy Survey Questionnaire (Abacıgil et al., 2019; Agrali and Akyar, 2018; Durusu Tanrıover et al., 2014; HLS-EU Consortium, 2012). Besides adaptation studies, there were also some initiatives to develop original measurement tools in Turkish (Sezer & Kadıoğlu, 2014). However, these studies were conducted with patient groups in primary care settings. Hence, despite numerous health literacy scales available in Turkish, none of the existing scales was originally developed and validated at a national level.

With this significant gap in mind, this study aimed to develop and validate a culturally sensitive original health literacy scale (HLS) to be used as a reference scale for the Turkish-speaking literate adult population in Turkey and abroad.

## Method

### Conceptualization of Health Literacy

***Step 1: Initial development of items.*** At the beginning, widely used international health literacy scales like REALM (Davis et al., 1991), TOFHLA (Parker et al., 1995), S-TOFHLA (Baker et al., 1999), NVS (Weiss et al., 2005), and adapted/locally developed scales in Turkey (Avcı, 2013; Durusu Tanrıover et al., 2014; Sezer & Kadıoğlu, 2014) were reviewed in terms of their scale-developing process and item structures. Then, to measure health literacy in different levels of cognitive domain and systemize the item development, a matrix was created. The horizontal axis of the matrix consisted of four categories (subdimensions) inspired from comprehensive public health approach related to health literacy (“disease prevention,” “health promotion,” “treatment,” “access to health services”). Vertical axis consisted of three categories (“knowledge,” “comprehension,” and “application”) of the cognitive domain of Bloom's taxonomy) (Herr, 2007). In this axis, knowledge was defined as “the remembering of previously learned material”; comprehension was defined as “the ability to grasp the meaning of material” and application referred to “the ability to use learned material in new and concrete situations” (Herr, 2007, p. 1) (**Table 1**). In addition, a subjective measurement part involving “self-efficacy” items were added for attitude and awareness (“affective domain” included feelings, values, appreciation, enthusiasms, motivations, and attitudes in Bloom's taxonomy) (Anderson, 2001). The headings and some samples of items for each category of the matrix were given in **Table 1**.

**Table 1:**
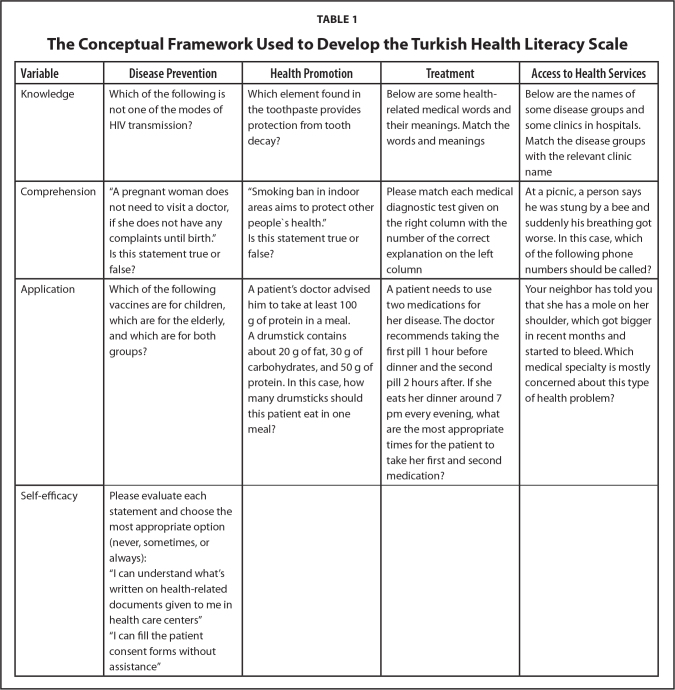
The Conceptual Framework Used to Develop the Turkish Health Literacy Scale

**Variable**	**Disease Prevention**	**Health Promotion**	**Treatment**	**Access to Health Services**
Knowledge	Which of the following is not one of the modes of HIV transmission?	Which element found in the toothpaste provides protection from tooth decay?	Below are some health-related medical words and their meanings. Match the words and meanings	Below are the names of some disease groups and some clinics in hospitals. Match the disease groups with the relevant clinic name
Comprehension	“A pregnant woman does not need to visit a doctor, if she does not have any complaints until birth.” Is this statement true or false?	“Smoking ban in indoor areas aims to protect other peoplès health.” Is this statement true or false?	Please match each medical diagnostic test given on the right column with the number of the correct explanation on the left column	At a picnic, a person says he was stung by a bee and suddenly his breathing got worse. In this case, which of the following phone numbers should be called?
Application	Which of the following vaccines are for children, which are for the elderly, and which are for both groups?	A patient's doctor advised him to take at least 100 g of protein in a meal. A drumstick contains about 20 g of fat, 30 g of carbohydrates, and 50 g of protein. In this case, how many drumsticks should this patient eat in one meal?	A patient needs to use two medications for her disease. The doctor recommends taking the first pill 1 hour before dinner and the second pill 2 hours after. If she eats her dinner around 7 pm every evening, what are the most appropriate times for the patient to take her first and second medication?	Your neighbor has told you that she has a mole on her shoulder, which got bigger in recent months and started to bleed. Which medical specialty is mostly concerned about this type of health problem?
Self-efficacy	Please evaluate each statement and choose the most appropriate option (never, sometimes, or always): “I can understand what's written on health-related documents given to me in health care centers” “I can fill the patient consent forms without assistance”			

Thereafter, a multidisciplinary workshop with 20 experts (from fields such as public health, family medicine, internal medicine, obstetrics and gynecology, nursing, educational measurement and evaluation, biostatistics, sociology, social work, journalism, communication, Turkish language and literature, Turkish folklore) was conducted. During this workshop, the experts developed a 404-item pool by using the abovementioned matrix (composed of multiple choice, true/false, and matching questions). In addition, 20 self-efficacy statements were added to the scale.

***Step 2: Reducing the items (first pre-test)***: The first draft of the measurement tool was pre-tested with 20 adults chosen haphazardly from different gender and age groups from a district of Ankara city. Then, in a second workshop with the same experts, the results of the pre-test were discussed. During this workshop, the items, which were not properly understood by the participants of the pre-test, were deleted and some were revised. At the end, the second draft of the scale was developed with 243 items plus 20 self-efficacy statements. There was no need to make any revision in self-efficacy statements.

***Step 3: Second pre-test***: A second pre-test was conducted with 150 adults from different age groups (between ages 18 and 60 years) and socioeconomic levels. The participants resided at one district of Ankara, and their age and gender distribution were similar to the final sample of the validation study. The data collected were analyzed by performing item-total correlation analysis. In the end, 110 items with ≥0.5 loading were used for the purpose of scale construction, and then the third draft scale was generated with 110 items plus 20 self-efficacy statements.

***Step 4: Validation study (community-based)***: The priority in scale development process is to examine the group that best predicts the properties of the items in the scale; it does not matter whether the sample represents the population or not. The information obtained from the scale to be developed is used to interpret the items in the scale, not to generalize to the population from which the sample is taken. From this point, authors suggest that the sample size should be a certain multiple of the number of items in the newly developed scale (Erkuş, 2012; Stevens, 1996), whereas some others stated that between 500 and 1,000 people are good or very good for the sample size (Comrey & Lee, 1996). In our study, based on the information above, the sample size was determined.

Turkey was hypothetically divided randomly into 12 Nomenclature of Territorial Units for Statistics (NUTS) according to different economic and cultural/social characteristics by the Turkish Statistical Institute. From each province, 200 adults were selected (with an equal number of men and women in age groups 18–29 years, 30–39 years, and 40–60 years), for a total of 2,400 participants by using convenience sampling to perform the validation study of the scale. The study was carried out with a household survey of 2,466 adults (in some provinces, 66 additional participants were recruited due to convenience). Data analysis was performed with 2,411 adults after removing 55 health worker participants.

**Figure 1.**
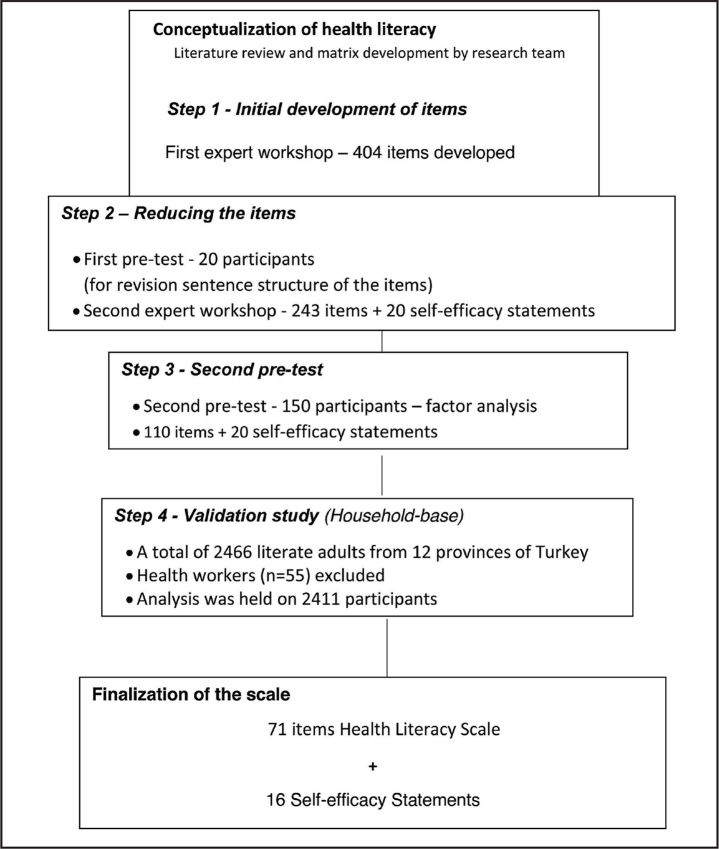
Study flow chart.

After performing the validity and reliability analysis, 71 items plus 16 self-efficacy statements remained for the final version of the scale. See **Figure 1** for the study flow chart.

### Statistical Analysis

Data analyses involved calculation of the discrimination index, mean and standard deviations for the items, as well as validity-reliability measures for the scale. Stratified Cronbach's alpha and McDonald's omega coefficients were used for the overall reliability, whereas Cronbach's alpha and split-half reliability statistics were used to test the reliability of the sub-test of the scale. Confirmatory factor analysis based on polychoric correlations was performed to show construct validity of two-dimensional scale. In addition, one-dimensional model and second order confirmatory factor analysis were applied, and the results were compared with two-dimensional model. Every item of the scale was scored as “1”: *true answer* and “0”: *wrong answer*. Self-efficacy statements were scored as “1”: *never*; “2”: *sometimes*; and “3”: *always*. SPSS version 23.0, R version 4.0.0, and FACTOR 10.8.02 programs were used for data analyses.

Ethical approval for the study was obtained from Hacettepe University Non-Interventional Clinical Researches Ethics Board, and official permission from the Ministry of Interior.

## Results

### The Analysis for Health Literacy Scale

***Item analysis:*** Public health and educational measurement and evaluation experts discussed the appropriateness and distribution of the items to Bloom's taxonomy after the second pre-test process again and made the necessary item revisions. After the implementation of the household survey, the compatibility of the expert opinions with the application results was checked by using the correlations between the items.

In this analysis, frequencies as well as difficulty and discrimination of items were evaluated. It was found that the items generally showed positive correlation. The difficulty levels of the items were ranged from 0.11 to 0.97 for knowledge, comprehension, and application dimensions, and 0.07 to 0.97 for disease prevention, health promotion, treatment, and access to health services subscales. According to the Bloom's taxonomy dimensions, all of the values of the correlation of items with the sum of other items in each dimension were positive. It has been observed that the correlations and expert opinions were compatible.

Items with negative item-total correlation and items with item-total correlation less than 0.20 were excluded from the scale. In addition to the statistical values, the effects of the items on the content validity of the scale as well as the four public health experts' opinions were also taken into consideration when removing the items from the scale. According to the results, four subscales, which can be seen in **Table 1**, were needed to be recombined into two subscales as “disease prevention-health promotion” and “treatment-access to health services.” After that, item analysis was re-performed for two-dimensional structure and the statistics were found to be sufficient. According to the item analysis results, it was observed that the difficulty values of the items ranged between 0.29 and 0.87 and the discrimination values (point-biserial correlations) varied between 0.21 and 0.45 for the “disease prevention-health promotion” dimension; these values were 0.20 and 0.97 and 0.09 and 0.56, respectively, for the “treatment-access to health services” dimension of the final scale, which consists of 71 items (from 110 items at the beginning of the analysis).

***Confirmatory factor analysis***: Construct validity study was conducted with confirmatory factor analysis (CFA) without performing exploratory factor analysis (EFA) for the two-dimensional health literacy scale because there was a theoretical basis for two-dimensionality.

In addition, the two-dimensional model was compared with one-dimensional and second order CFA models. According to the results, one-dimensional and two-dimensional models demonstrated a very good fitness. The fit indices were quite similar. The second order CFA model also showed a good fit; however, the two-dimensional model had the best fit indices compared to others. The results of the CFA analysis performed to confirm the two-dimensionality of the scale where all indices were found to meet the criteria very well, except “Chi-square/degree of freedom.” Because the chi-square test value has been found to be very high when the sample size is more than 200 participants, the results of chi-square goodness of fit were ignored (Kline, 2011). On the other hand, the construct validity of the scale was satisfied according to the other fit indices (**Table 2**). The correlation between the factor scores of the two dimensions (disease prevention-health promotion and treatment-access to health services) was 0.90 (*p* < .001). This value also gave a strong idea of the additivity of the two dimensions. These results showed that the total scores and sub-scale scores could be used together.

**Table 2:**
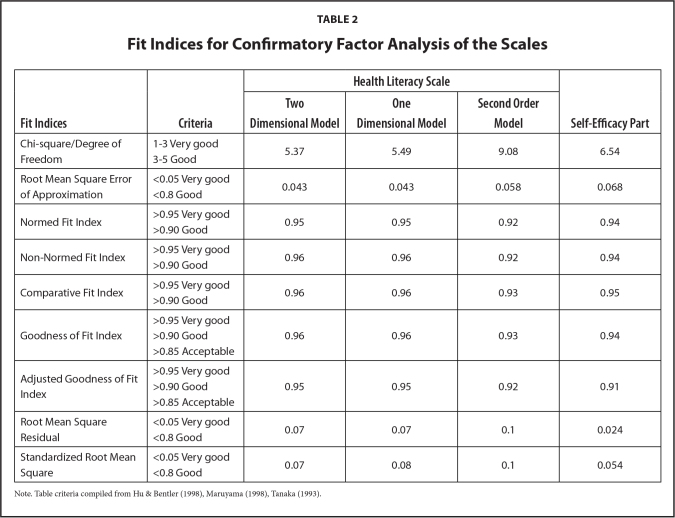
Fit Indices for Confirmatory Factor Analysis of the Scales

**Fit Indices**	**Criteria**	**Health Literacy Scale**			**Self-Efficacy Part**

		**Two Dimensional Model**	**One Dimensional Model**	**Second Order Model**	
Chi-square/Degree of Freedom	1–3 Very good	5.37	5.49	9.08	6.54
	3–5 Good				

Root Mean Square Error of Approximation	<0.05 Very good	0.043	0.043	0.058	0.068
	<0.8 Good				

Normed Fit Index	>0.95 Very good	0.95	0.95	0.92	0.94
	>0.90 Good				

Non-Normed Fit Index	>0.95 Very good	0.96	0.96	0.92	0.94
	>0.90 Good				

Comparative Fit Index	>0.95 Very good	0.96	0.96	0.93	0.95
	>0.90 Good				

Goodness of Fit Index	>0.95 Very good	0.96	0.96	0.93	0.94
	>0.90 Good				
	>0.85 Acceptable				

Adjusted Goodness of Fit Index	>0.95 Very good	0.95	0.95	0.92	0.91
	>0.90 Good				
	>0.85 Acceptable				

Root Mean Square Residual	<0.05 Very good	0.07	0.07	0.1	0.024
	<0.8 Good				

Standardized Root Mean Square	<0.05 Very good	0.07	0.08	0.1	0.054
	<0.8 Good				

Note. Table criteria compiled from Hu & Bentler (1998), Maruyama (1998), Tanaka (1993).

***Reliability analysis***: Cronbach's alpha and Guttman Lambda-2 reliability coefficients calculated for the sub-dimensions of the scale were high. The split-half reliability coefficient of “disease prevention-health promotion” dimension (Cronbach's alpha = 0.785, split-half reliability = 0.662, Guttman Lambda-2 = 0.792) was acceptable and “treatment-access to health services” subdimension (Cronbach's alpha = 0.914, split-half reliability = 0.779, Guttman Lambda-2 = 0.917) was high. In addition, stratified alpha (0.93) and McDonald's omega coefficients (0.81) for the scale were quite high.

### The Analysis for Self-Efficacy Part

Self-efficacy part of the scale was designed as one-dimensional instrument, and EFA, CFA and reliability analyses were performed. The data were randomly divided into two parts to perform EFA and CFA. For the first half of the data EFA, for the second half of the data CFA were applied to confirm the factor structure of the scale (both analyses were based on polychoric correlations). Reliability analysis was performed on the whole dataset.

The Kaiser-Meyer-Olkin (KMO) sampling adequacy test (Kaiser, 1970; 1974) showed that sample size was adequate for EFA (0.91). The Bartlett's Test of Sphericity, which tests whether the correlation matrix differs significantly from the identity matrix, was significant (*p* < .001). According to this result, data were suitable for explanatory factor analysis. EFA analysis was completed in two steps. In the first step, 20 statements were analyzed. In the second step, the analysis was repeated after excluding 4 statements removed from the scale due to the low factor loads. The results of parallel analysis showed that one-dimensional structure was supported. After EFA was repeated, the results showed that KMO value was 0.90, indicating that the sample size was sufficient. Similarly, the Bartlett's Test of Sphericity was statistically significant (*p* < .001), which showed that the correlation between the variables was sufficient to perform factor analysis

**Figure 2.**
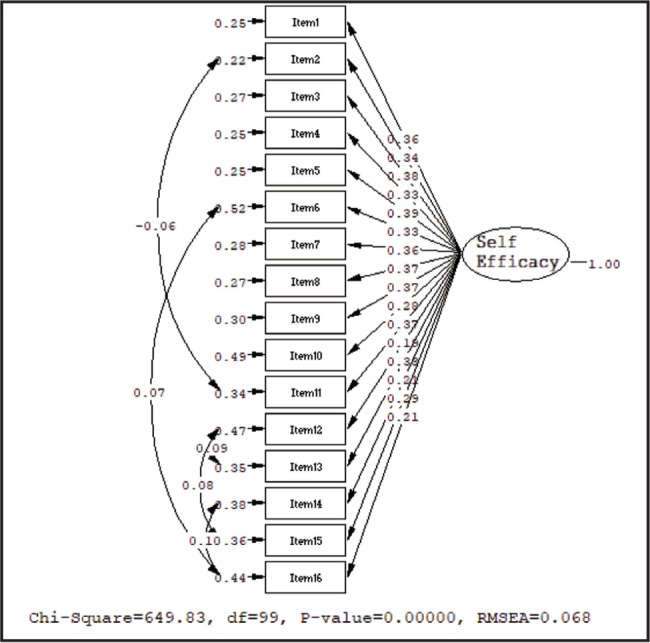
The path diagram of the self-efficacy part.

Unweighted least squares method, one of the most suitable methods for the non-normally distributed data, was used as an estimation method in CFA. The path graph, which shows the standardized factor loadings of the self-efficacy part, was given in **Figure 2**.

According to the path coefficients, there were some modifications between some of the statements. In the process of making the modifications, the following were taken into consideration: error variances, being related items in one-dimension, low number of modifications, and significant increase in chi-square goodness of fit value. For the evaluation of this model, all the fit indices except “Chi-square/degree of freedom” met the model fit criteria at a good level (**Table 2**). The results of other fit indices obtained for the one-dimensional model provide important evidence for the construct validity of the self-efficacy part.

### The Reliability Analysis of Self-Efficacy Part

Split-half reliability, Cronbach's Alpha, Guttman Lambda 2 and McDonald's Omega coefficients were calculated to evaluate the reliability of the self-efficacy part. Cronbach's alpha (0.83), McDonald's omega (0.88), Gutman Lambda-2 (0.83), and split-half reliability (0.73) coefficients were higher than 0.70. These results showed that the self-efficacy scale was quite reliable.

### Criterion Validity

It was expected that health workers would have a higher health literacy score from others. From this point, the scores of 55 health workers not included in the analysis were used to assess the criterion validity of the newly developed HLS. From the study group of 2,411 non-health worker participants, 55 were randomly selected in the same age and gender groups with the health worker participants. The mean scores of the scale and self-efficacy part of health-worker and non-health worker participants were compared; the difference was statistically significant (**Table 3**); hence, the criterion validity of the scale was confirmed.

**Table 3:**
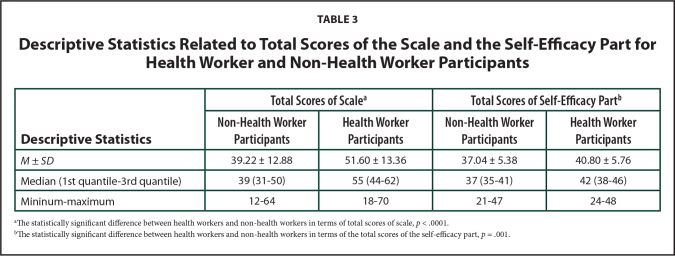
Descriptive Statistics Related to Total Scores of the Scale and the Self-Efficacy Part for Health Worker and Non-Health Worker Participants

**Descriptive Statistics**	**Total Scores of Scale^a^**		**Total Scores of Self-Efficacy Part^b^**	
	**Non-Health Worker Participants**	**Health Worker Participants**	**Non-Health Worker Participants**	**Health Worker Participants**
*M* ± *SD*	39.22 ± 12.88	51.60 ± 13.36	37.04 ± 5.38	40.80 ± 5.76
Median (1st quantile-3rd quantile)	39 (31–50)	55 (44–62)	37 (35–41)	42 (38–46)
Mininum–maximum	12–64	18–70	21–47	24–48

a The statistically significant difference between health workers and non-health workers in terms of total scores of scale, *p* < .0001.

b The statistically significant difference between health workers and non-health workers in terms of the total scores of the self-efficacy part, *p* = .001.

## Discussion

Improving health literacy has become one of the most important health-related goals at the global level. The World Health Organization (2008) emphasizes that health literacy is one of the fundamental determinants of health and that all countries need to monitor and improve health literacy levels in a continuous manner. Low health literacy is consistently associated with poorer ability to interpret health messages, higher frequency of risky health behaviors, inappropriate use of health services, more hospitalizations, and poorer performance on rational medicine use (Berkman et al., 2011; Howard et al., 2005; Seldon et al., 2000).

Health literacy scales are important tools for assessing the health literacy of populations for planning and evaluating evidence-based health education and promotion interventions. However, there are significant differences in how health literacy is defined, and which dimensions are taken into consideration regarding this concept (Poureslami et al., 2017; Sørensen et al., 2012). Several articles emphasized that health literacy involves a combination of skills including the ability to interpret documents, read and write prose (print literacy), use quantitative information (numeracy or quantitative literacy), as well as being able to communicate effectively (oral literacy) (Altin et al., 2014; Berkman et al., 2010). On the other hand, one of the most widely discussed approaches to literacy classification included three levels: namely basic/functional literacy, communicative/interactive literacy, and critical literacy. This comprehensive approach indicated that the different levels of literacy progressively allowed for greater autonomy and personal empowerment (Nutbeam, 2000; 2008). For instance, TOFHLA consists of two parts and assesses the reading comprehension and numeracy (Parker, 1995); REALM is a quick screening tool identifying the patient reading levels (Davis et al., 1991); REALM-R is a word recognition test only (Bass et al, 2003), and NVS test, measures reading and interpretation skills (Weiss et al., 2005). However, revealed from the comprehensive approach, in our HLS, the three levels of cognitive domain of Bloom's Taxonomy (“knowledge,” “comprehension,” and “application”) were used to develop the items as well as the self-efficacy statements, which are based on the affective domain.

There were some other adopted health literacy scales existing in Turkey (Abacıgil et al., 2019; Durusu Tanrıover et al., 2014; Ozdemir et al., 2010). However, there was no original scale developed for healthy adults, representing the country and developed in its own language. This is the first Turkish original scale of its kind. Our findings showed that HLS is both a valid and reliable measurement tool to assess health literacy of Turkish speaking, literate adults.

The process of adapting a measurement tool to another language was seen primarily as a linguistic task in the past. Currently, when a measurement tool is used in different cultures and languages, cultural adaptation as well as preserving its linguistic content accepted. However, it is seen that cultural elements are not sufficiently included in adaptation studies. The large use of a scale in western cultures does not necessarily mean that it will fit well into Turkish culture (Çapık et al, 2018). The adaptation term refers transferring the scale from one language to another (International Test Commission [ITC], 2018). Very faithful, very free, or very literary translations of the original text may not be culturally appropriate and make it difficult to achieve equality in two languages (Çapık et al., 2018). Differences in language structure can cause problems in test translation. For example, the fill in the blanks format is inappropriate in the Turkish language, where the object of a sentence must come before the verb and subject. Because the Turkish people should first look at the end of the statement before they fill out the beginning, the use of incomplete sentences in the English versions of health literacy scales would change the answering behavior completely (ITC, 2018). Two different questions from the TOFHLA scale are given as examples of this reality: “I . . . to provide the county information to . . . any statements given in this . . . and hereby give permission to the . . . to get such proof I . . . that for Medicaid I must report any . . . in my circumstances within . . . (10) days of becoming . . . of the change”; “You must have an . . . stomach when you come for . . .” (McWhorter, 2019). In this regard, if the order of use of the subject and verb in Turkish and English is not considered in translation, there might be misunderstandings (ITC, 2018). Weiss et al. (2005) experienced a similar problem in their study of NVS, which the original language is English. According to the authors, “the psychometric properties of the Spanish version of the NVS, were not as good as those of the English version. This fact may stem from the greater heterogeneity of language and culture among Spanish-speaking patients” (Weiss et al., 2005, p. 521).

Our original HLS was developed in a linguistically and culturally appropriate manner with the contribution of 20 experts from fields of public health, family medicine, internal medicine, obstetrics and gynecology, nursing, educational sciences, biostatistics, sociology, social work, journalism, communication, Turkish language and literature, and Turkish folklore. This combination of experts had ensured both the appropriateness of cultural and Turkish grammar structure as well as the health dimension.

For the development process of TOFHLA, REALM, and NVS, the validation studies performed on patients admitted to outpatient clinics of hospitals or public and private primary care clinics (Davis et al., 1991; Parker et al., 1995; Weiss et al., 2005). However, as it is well known, some patients could have special characteristics and present specific health literacy levels. For this reason, it is important to develop a scale for determining the level of health literacy of a healthy general population. In our study, the data were gathered at the national level via a household study from healthy people, which were covered in 12 randomly selected provinces of 81 from every NUTS in Turkey. Working with a community-based sample by visiting households, rather than working with specific patient or service user groups in health care settings, as well as the application of the scale to a geographically and culturally diverse population are among the other significant strengths of our original scale.

In conclusion, we suggest that different research groups can use this original HLS to assess the health literacy level of Turkish speaking adults living in Turkey and abroad. The scale will enable standard monitoring, evaluation, and comparison of health literacy levels among different adult population groups; the scale will also assess the effect of health education and health promotion policies and programs in the long term. Even though the scale was developed for Turkish-speaking adults, we believe that the scale can also be adapted to similar languages and cultures in the Eastern Europe and Central Asia region.

## Study Limitations

The current study had several limitations. Study participants were literate adults age 18 to 60 years. For this reason, it may not be appropriate to use this scale for adolesecents or older adults without validity and reliability studies. Because the scale is self-administered, it may not be suitable for people with very low educational attainment. Even though the participants were recruited from different regions of Turkey, the sampling method (convenience sampling) and related external validity may have created additional limitations.

## References

[x24748307-20211208-01-bibr1] Abacigil , F. , Harlak , H. , Okyay , P. , Kiraz , D. E. , Gursoy Turan , S. , Saruhan , G. , Karakaya , K. , Tuzun , H. , Baran Deniz , E. , Tontus , O. , & Beşer , E. ( 2019 ). Validity and reliability of the Turkish version of the European Health Literacy Survey Questionnaire . *Health Promotion International* , *34* ( 4 ), 658 – 667 . 10.1093/heapro/day020 PMID: 29648593

[x24748307-20211208-01-bibr2] Agrali , H. , & Akyar , I. ( 2018 ). Turkish validation and reliability of health literacy scale for diabetic patient . *ACU Saglik Bil Derg* , *9* ( 3 ), 314 – 321 .

[x24748307-20211208-01-bibr3] Altin , S. V. , Finke , I. , Kautz-Freimuth , S. , & Stock , S. ( 2014 ). The evolution of health literacy assessment tools: A systematic review . *BMC Public Health* , *14* ( 1207 ), 1207 10.1186/1471-2458-14-1207 PMID: 25418011 PMC4289240

[x24748307-20211208-01-bibr4] Anderson , L.W. , Krathwohl , D.R. , Airasian , P.W. , Cruikshank , K.A. , Mayer , R.E. , Pintrich , P.R. , Raths , J. , Wittrock , M.C. ( 2001 ). *A taxonomy for learning, teaching, and assessing: a revision of bloom's taxonomy of educational objectives* . Pearson, Allyn & Bacon .

[x24748307-20211208-01-bibr5] Avcı , E . ( 2013 ). Annelerin anne sütü ile ilgili sağlık okuryazarlık düzeylerini değerlendirme aracı geliştirme [Developing breastfeeding health literacy measuring instrument for mothers, evaluating the levels of mothers' health literacy about breastfeeding with this instrument and determining the factors that affects these factors]. Sağlık okuryazarlık düzeylerini ve etkileyen faktörleri saptama [Unpublished medical specialty thesis] . Gazi University, Department of Medicine, Department of Public Health .

[x24748307-20211208-01-bibr6] Baker , D. W. , Williams , M. V. , Parker , R. M. , Gazmararian , J. A. , & Nurss , J. ( 1999 ). Development of a brief test to measure functional health literacy . *Patient Education and Counseling* , *38* ( 1 ), 33 – 42 . 10.1016/S0738-3991(98)00116-5 PMID: 14528569

[x24748307-20211208-01-bibr7] Bass , P. F. III , Wilson J. F. , & Griffith , C. H. ( 2003 ). A shortened instrument for literacy screening . *Journal of General Internal Medicine* , *18* ( 12 ), 1036 – 1038 . 10.1111/j.1525-1497.2003.10651.x PMID: 14687263 PMC1494969

[x24748307-20211208-01-bibr8] Berkman , N. D. , Davis , T. C. & McCormack , L. ( 2010 ). Health literacy: What is it? *Journal of Health Communication* , *15* ( Suppl. 2 ), 9 – 19 . 10.1080/10810730.2010.499985 20845189

[x24748307-20211208-01-bibr9] Berkman , N. D. , Sheridan , S. L. , Donahue , K. E. , Halpern , D. J. , & Crotty , K. ( 2011 ). Low health literacy and health outcomes: An updated systematic review . *Annals of Internal Medicine* , *155* ( 2 ), 97 – 107 . 10.7326/0003-4819-155-2-201107190-00005 PMID: 21768583

[x24748307-20211208-01-bibr10] Çapık , C. , Gözüm , S. , & Aksayan , S. ( 2018 ). Kültürlerarası Ölçek Uyarlama Aşamaları, Dil ve Kültür Uyarlaması: Güncellenmiş Rehber [Intercultural scale adaptation stages, language and culture adaptation: updated guideline] . *FNJN Florence Nightingale Journal of Nursing* , *26* ( 3 ), 199 – 210 . 10.26650/FNJN397481

[x24748307-20211208-01-bibr11] Comrey , A. L. , & Lee , H. B. ( 1992 ). *A first course in factor analysis* ( 2nd ed. ). Lawrence Erlbaum Associates, Publishers, Hillsdale .

[x24748307-20211208-01-bibr12] Curtis , L. M. , Revelle , W. , Waite , K. , Wilson , E. A. , Condon , D. M. , Bojarski , E. , Park , D. C. , Baker , D. W. , & Wolf , M. S. ( 2015 ). Development and validation of the comprehensive health activities scale: A new approach to health literacy measurement . *Journal of Health Communication* , *20* ( 2 ), 157 – 164 . 10.1080/10810730.2014.917744 PMID: 25375025 PMC4346471

[x24748307-20211208-01-bibr13] Davis , T. C. , Crouch , M. A. , Long , S. W. , Jackson , R. H. , Bates , P. , George , R. B. , & Bairnsfather , L. E. ( 1991 ). Rapid assessment of literacy levels of adult primary care patients . *Family Medicine* , *23* ( 6 ), 433 – 435 PMID: 1936717

[x24748307-20211208-01-bibr14] Durusu Tanrıöver M. , Yıldırım , H.H. , Demiray Ready F. N. , Çakır B. , & Akalın H. E. ( 2014 ). *Turkish Health Literacy Survey (SOYA)* . Sağlık-Sen Yayınları .

[x24748307-20211208-01-bibr15] Erkuş , A. ( 2012 ). *Psikolojide Ölçme ve Ölçek Geliştirme-I: Temel Kavramlar ve İşlemler [Measurement and scale development in psychology-i: basic terms and applications]* . Pegem Akademi Yayınları .

[x24748307-20211208-01-bibr16] Eyüboğlu E. , & Schulz P. J. ( 2016 ). Validation of Turkish health literacy measures . *Health Promotion International* , *31* ( 2 ), 355 – 362 . 10.1093/heapro/dau111 PMID: 25586111

[x24748307-20211208-01-bibr17] Fransen , M. P , Van Schaik , T. M. , Twickler , T. B. & Essink-Bot , M. L. ( 2011 ). Applicability of internationally available health literacy measures in the Netherlands . *Journal of Health Communication* , *16* ( Suppl. 3 ), 134 – 149 . 10.1080/10810730.2011.604383 21951248

[x24748307-20211208-01-bibr18] Herr , N . ( 2007 ). *The sourcebook of teaching science: Bloom's taxonomy* . https://www.csun.edu/science/ref/reasoning/questions_blooms/blooms.html#Knowledge

[x24748307-20211208-01-bibr19] HLS-EU Consortium . ( 2012 ). *Comparative report on health literacy in eight EU member states* . http://cpme.dyndns.org:591/adopted/2015/Comparative_report_on_health_literacy_in_eight_EU_member_states.pdf

[x24748307-20211208-01-bibr20] Howard , D. H. , Gazmararian , J. , & Parker , R. M. ( 2005 ). The impact of low health literacy on the medical costs of Medicare managed care enrollees . *The American Journal of Medicine* , *118* ( 4 ), 371 – 377 . 10.1016/j.amjmed.2005.01.010 PMID: 15808134

[x24748307-20211208-01-bibr21] Hu , L. T. , & Bentler , P. M. ( 1998 ). Fit indices in covariance structure modeling: Sensitivity to underparameterized model misspecification . *Psychological Methods* , *3* ( 4 ), 424 – 453 . 10.1037/1082-989X.3.4.424

[x24748307-20211208-01-bibr22] International Test Commission ( 2018 ). ITC guidelines for translating and adapting tests . *International Journal of Testing* , *18* ( 2 ), 101 – 134 . 10.1080/15305058.2017.1398166

[x24748307-20211208-01-bibr23] Kaiser , H. F. ( 1970 ). A Second Generation Little Jiffy . *Psychometrika* , *35* ( 4 ), 401 – 415 . 10.1007/BF02291817

[x24748307-20211208-01-bibr24] Kaiser , H. F. ( 1974 ). An Index of Factorial Simplicity . *Psychometrika* , *39* ( 1 ), 31 – 36 . 10.1007/BF02291575

[x24748307-20211208-01-bibr25] Kline , R. B. ( 2011 ). *Principles and practice of structural equation modeling* ( 3rd ed .). Guilford Press .

[x24748307-20211208-01-bibr26] Maruyama , G. M. ( 1998 ). *Basics of structural equation modeling* . Sage Publications . 10.4135/9781483345109

[x24748307-20211208-01-bibr27] McWhorter , A . ( 2019 ). Health literacy and health information seeking behaviors of student at the University of Central Florida (Publication No. 636) [Honors undergraduate thesis , University of Central Florida ]. https://stars.library.ucf.edu/honorstheses/636

[x24748307-20211208-01-bibr28] Nutbeam , D. ( 2000 ). Health literacy as a public health goal: A challenge for contemporary health education and communication strategies into the 21st century . *Health Promotion International* , *15* ( 3 ), 259 – 267 . 10.1093/heapro/15.3.259

[x24748307-20211208-01-bibr29] Nutbeam , D. ( 2008 ). The evolving concept of health literacy . *Social Science & Medicine* , *67* ( 12 ), 2072 – 2078 . 10.1016/j.socscimed.2008.09.050 PMID: 18952344

[x24748307-20211208-01-bibr30] Ozdemir , H. , Alper , Z. , Uncu , Y. , & Bilgel , N. ( 2010 ). Health literacy among adults: A study from Turkey . *Health Education Research* , *25* ( 3 ), 464 – 477 . 10.1093/her/cyp068 PMID: 20080808

[x24748307-20211208-01-bibr31] Parker , R. M. , Baker , D. W. , Williams , M. V. , & Nurss , J. R. ( 1995 ). The test of functional health literacy in adults: A new instrument for measuring patients' literacy skills . *Journal of General Internal Medicine* , *10* ( 10 ), 537 – 541 . 10.1007/BF02640361 PMID: 8576769

[x24748307-20211208-01-bibr32] Pleasant, A., McKinney, J., & Rikard R. V. (2011) Health literacy measurement: A proposed research agenda. *Journal of Health Communication*, *16*(Suppl. 3), 11–21. 10.1080/10810730.2011.604392 21951240

[x24748307-20211208-01-bibr33] Poureslami , I. , Nimmon , L. , Rootman , I. , & Fitzgerald , M. J. ( 2017 ). Health literacy and chronic disease management: Drawing from expert knowledge to set an agenda . *Health Promotion International* , *32* ( 4 ), 743 – 754 PMID: 26873913 10.1093/heapro/daw003 PMC5914455

[x24748307-20211208-01-bibr34] Seldon , C. R. , Zorn , M. , Ratzan , S. , & Parker , R. M. ( 2000 ). *National Library of Medicine. Current bibliographies in medicine: Health literacy* . National Institutes of Health .

[x24748307-20211208-01-bibr35] Sezer , A. , & Kadıoğlu , H. ( 2014 ). Yetişkin Sağlık Okuryazarlığı Ölçeğinin Geliştirilmesi [Development of adult health literacy scale] . *Anadolu Hemsirelik ve Saglik Bilimleri Dergisi* , *17* ( 3 ), 165 – 170 .

[x24748307-20211208-01-bibr36] Sørensen , K. , Van den Broucke , S. , Fullam , J. , Doyle , G. , Pelikan , J. , Slonska , Z. , Brand , H. , & the (HLS-EU) Consortium Health Literacy Project European . ( 2012 ). Health literacy and public health: A systematic review and integration of definitions and models . *BMC Public Health* , *12* ( 80 ), 80 10.1186/1471-2458-12-80 PMID: 22276600 PMC3292515

[x24748307-20211208-01-bibr37] Stevens , J . ( 1996 ). *Applied multivariate statistics for the social sciences* ( 3rd ed. ). Erlbaum .

[x24748307-20211208-01-bibr38] Tanaka , J. S. ( 1993 ). Multifaceted conceptions of fit in structural equation models . In Bollen K. A. & Long J. S. (Eds.), *Testing structural equation models* (pp. 10 – 40 ). Sage .

[x24748307-20211208-01-bibr39] Weiss , B. D. , Mays , M. Z. , Martz , W. , Castro , K. M. , DeWalt , D. A. , Pignone , M. P. , Mockbee , J. , & Hale , F. A. ( 2005 ). Quick assessment of literacy in primary care: The newest vital sign . *Annals of Family Medicine* , *3* ( 6 ), 514 – 522 . 10.1370/afm.405 PMID: 16338915 PMC1466931

[x24748307-20211208-01-bibr40] World Health Organization . ( 2008 ). *Closing the gap in a generation: health equity through action on the social determinants of health - Final report of the commission on social determinants of health* . https://www.who.int/publications/i/item/WHO-IER-CSDH-08.1 10.1016/S0140-6736(08)61690-6 18994664

